# Supplementary material for: The Marine Dinoflagellate Alexandrium minutum Activates a Mitophagic Pathway in Human Lung Cancer Cells
Source: Mar Drugs. 2018 Dec 12;16(12):502. doi: 10.3390/md16120502 (PMC6316568; doi:10.3390/md16120502)
Supplement: Supplementary file 1 [file marinedrugs-16-00502-s001.pdf]

The marine dinoflagellate *Alexandrium minutum* activates a mitophagic pathway in human lung cancer cells

Christian Galasso[1]*, Genoveffa Nuzzo[2], Christophe Brunet[1]*, Adrianna Ianora[1], Angela Sardo[2], Angelo Fontana[2] and Clementina Sansone[1]

[1] Stazione Zoologica Anton Dohrn, Istituto Nazionale di Biologia, Ecologia e Biotecnologie marine, Villa Comunale, Naples, 80121, Italy;

[2] Bio-Organic Chemistry Unit, Institute of Biomolecular Chemistry-CNR, Via Campi Flegrei 34, Pozzuoli, Naples, 80078, Italy

* Correspondence and requests for materials should be addressed to C.G. and C.B.

(email: christian.galasso@szn.it; christophe.brunet@szn.it)

SUPPLEMENTARY INFORMATION

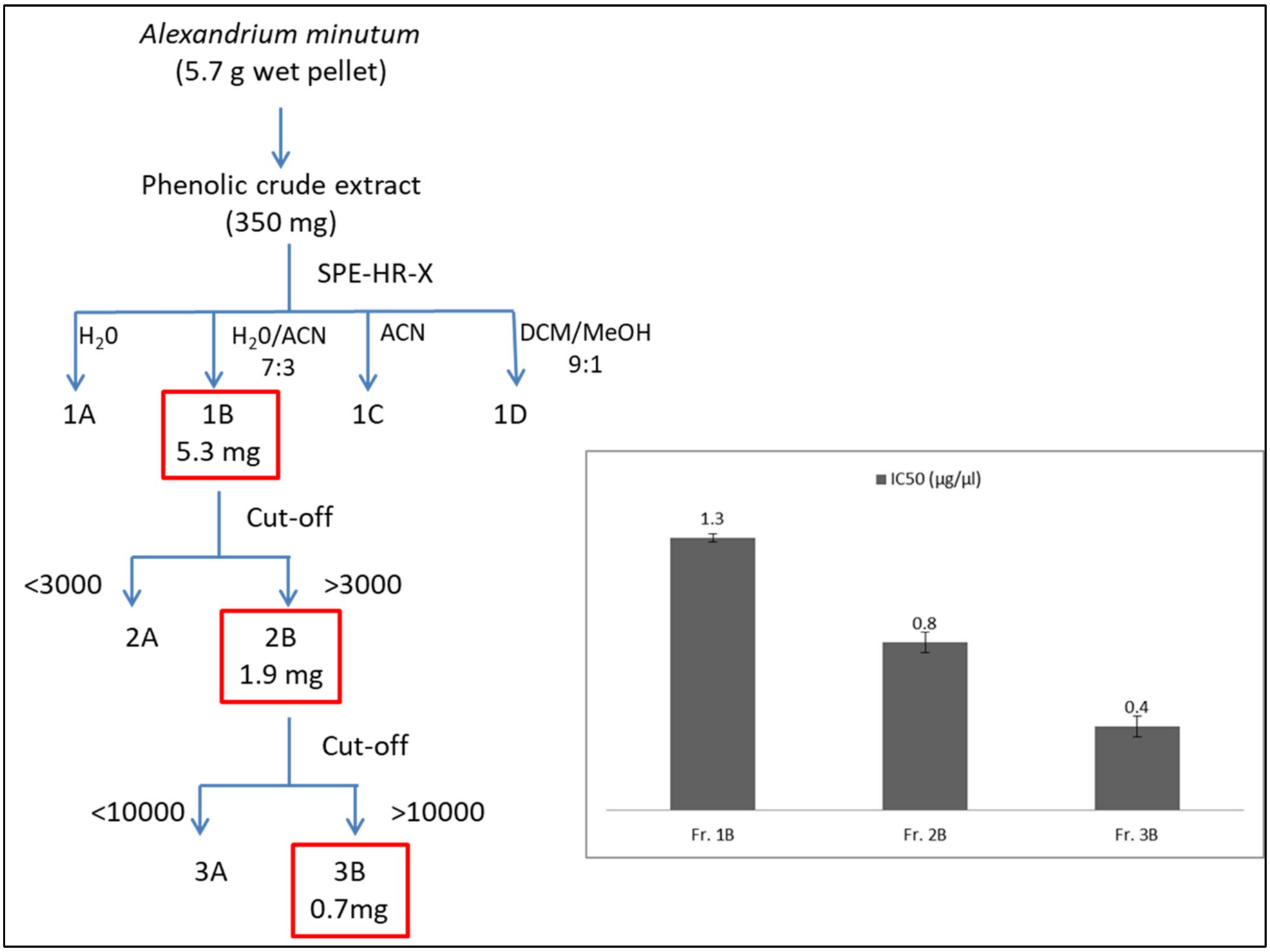

Figure S1. Bioassay-guided fractionation of *A. minutum* on lung adenocarcinoma cells A549. Bioactivity of fractions (1B, 2B and 3B) is reported as half maximal inhibitory concentration (IC50).

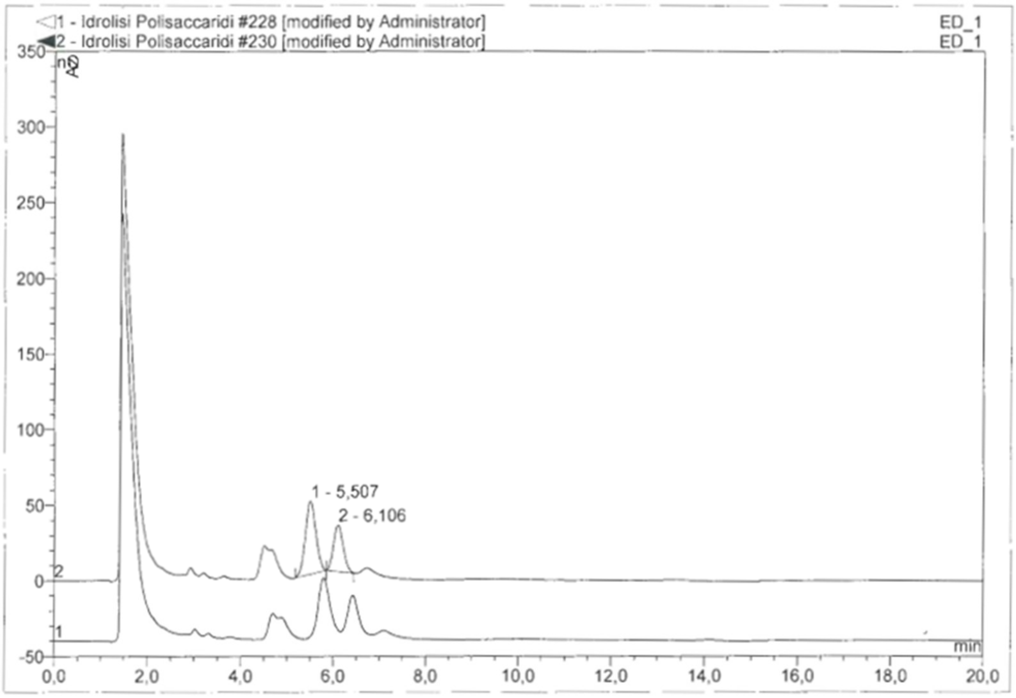

No.	Ret.Time min	Peak Name	Height nC	Area nC*min	Rel.Area %	Amount	Type
1	5,51	n.a.	48,239	12,725	63,36	n.a.	BMB*
2	6,11	n.a.	30,616	7,357	36,64	n.a.	BMB*
Total:			78,855	20,082	100,00	0,000	

Figure S2. High-performance anion-exchange chromatography (HPAEC) of hydrolyzed sugars (1 = D-galactose; 2 = D-glucose).

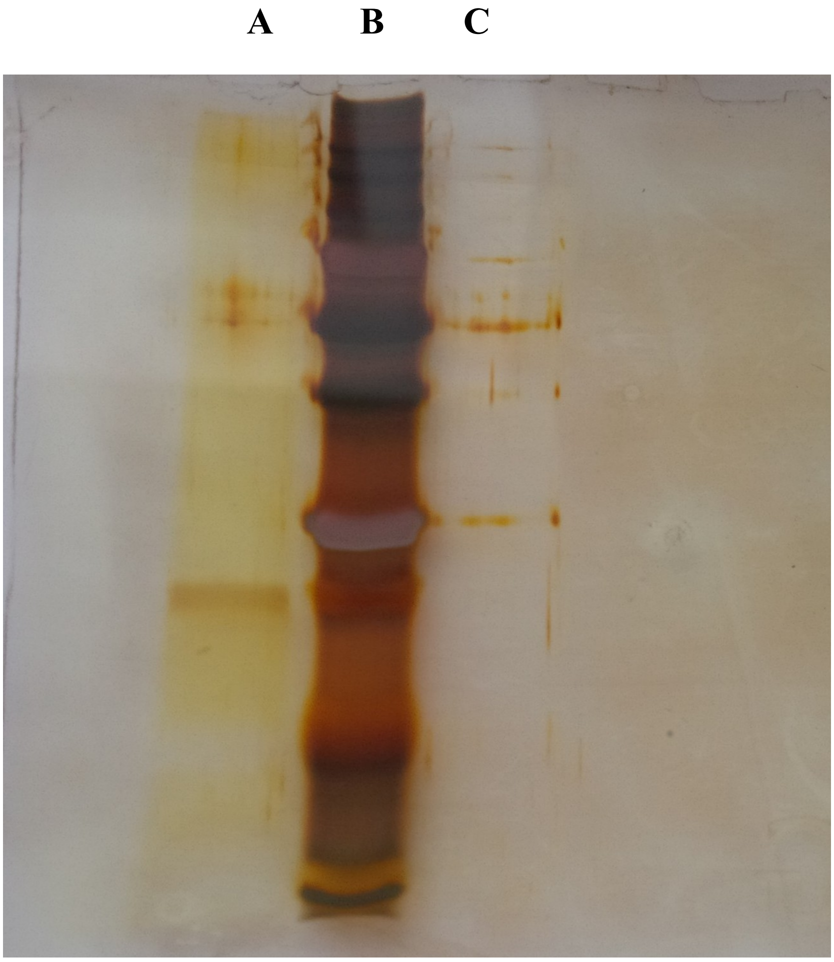

Figure S3. Whole electrophoresis gel of: A) fraction 3B (active sample); B) STD; C) fraction 4B (deglycosylated 3B sample).

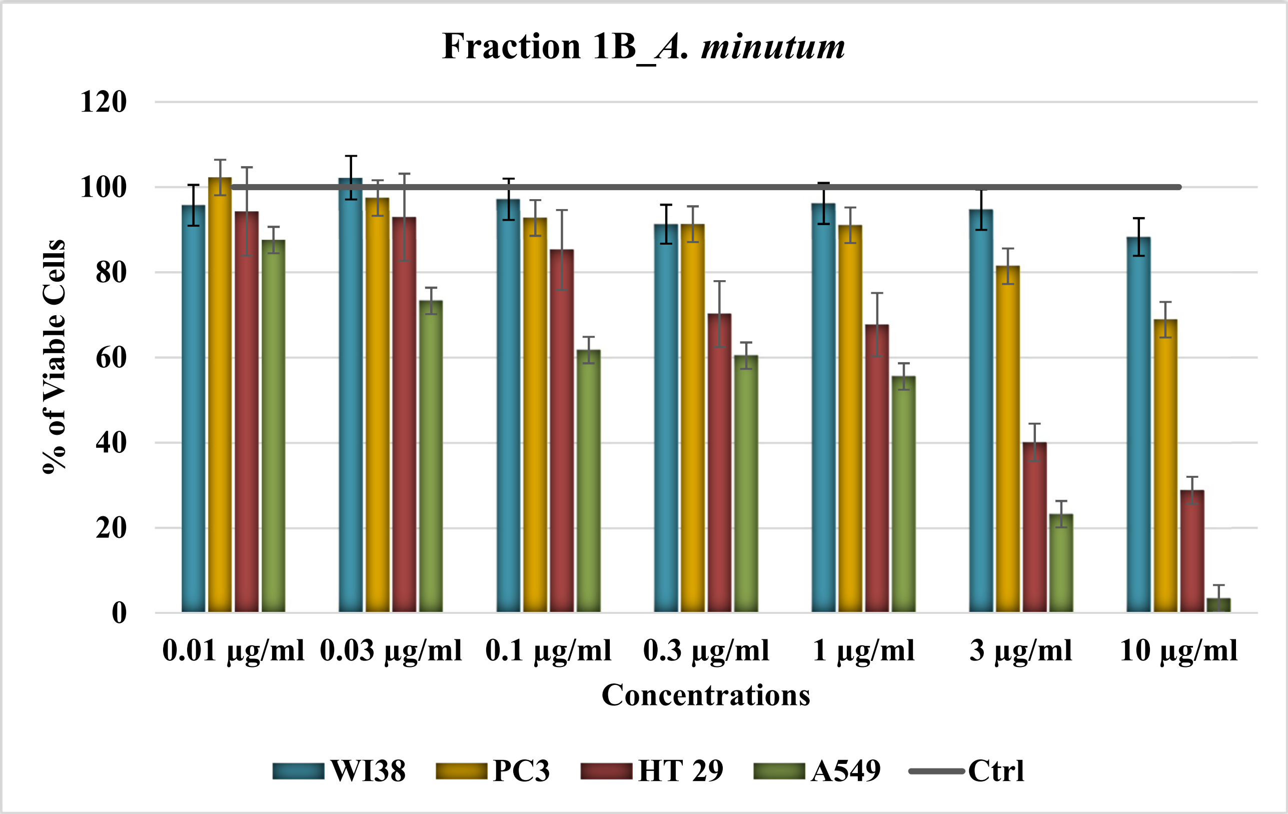

Figure S4. Effect of the fraction 1B on cell viability of human normal lung fibroblasts (WI38), human prostate cancer cells (PC3), human colorectal adenocarcinoma cells (HT29) and human lung adenocarcinoma cells (A549). Values are reported as mean ± S.D. compared to controls (100% viability) of three independent experiments. Concentrations tested were 0.01, 0.03, 0.1, 0.3, 1, 3 and 10 µg ml^{-1} for 48 h.

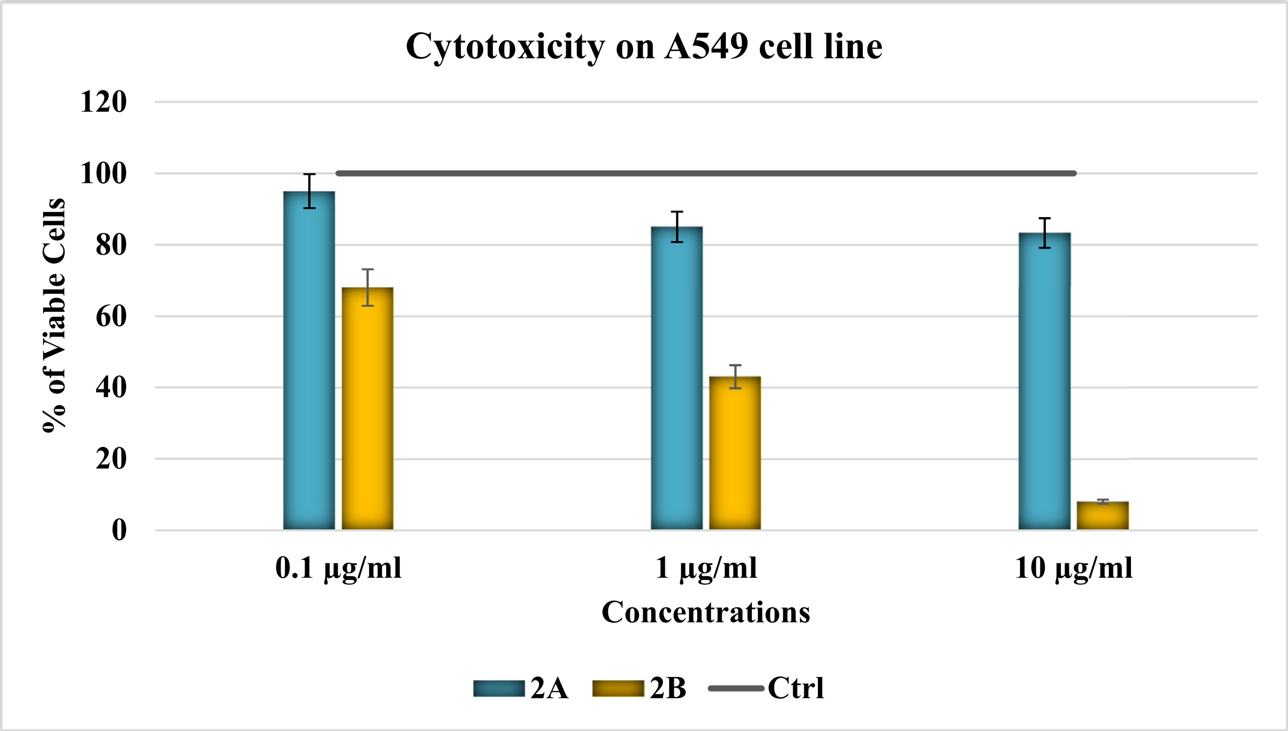

Figure S5. Effect of the fractions 2A and 2B on cell viability of human lung adenocarcinoma cells (A549). Values are reported as mean ± S.D. compared to controls (100% viability) of three independent experiments. Concentrations tested were 0.1, 1 and 10 µg ml^{-1} for 48 h.
